# 103 Beyond the Burn: Evaluating Insurance Support for Cosmetic Reconstruction After Burn Injury

**DOI:** 10.1093/jbcr/iraf019.103

**Published:** 2025-04-01

**Authors:** Anastasiya Ivanko, Athena Hoppe, Jonathan Schoen, Victoria Miles, Herbert Phelan, Jeffrey Carter

**Affiliations:** Burn Center at University Medical Center; Louisiana State University Health Sciences Center - New Orleans; Louisiana State University Health Sciences Center - New Orleans; University Medical Center New Orleans; Burn Center at University Medical Center; University Medical Center New Orleans

## Abstract

**Introduction:**

Patients recovering from burn injuries often encounter significant long-term psychological, physiologic, and biomechanical challenges related to scarring. Interventions that address these challenges while also enhancing self-esteem and quality of life exist; but, insurance coverage for them is often limited. This study examines the insurance policies of major health insurers regarding cosmetic reconstruction (CR) following burn injuries. burn injuries.

**Methods:**

A comprehensive review of coverage policies was conducted for five major health insurance providers: UnitedHealth Group, Elevance Health (formerly Anthem), Humana, CVS (Aetna), and Centene Corp. Reviews were conducted by ABA-verified burn center research staff focusing on invasive and noninvasive interventions for CR, trauma, and/or burn injury. Data was collected using Microsoft Excel® for statistical analysis from state-specific insurance documentation to determine the availability of coverage for CR post-burn injury.

**Results:**

In 2024, UnitedHealth Group holds 28% of the Medicare Advantage market (MAM) and 14% of the commercial markets (CM), operating in all 50 states. Humana represents 18% of the MAM and serves all 50 states. CVS (Aetna) has an 11% share in both CM and MAM, also covering all states. While these three major insurers have policies regarding CR post-burn injury, none provide coverage unless there is documented significant functional impairment (FI). Specifically, coverage for laser treatments aimed at reducing scar visibility is only available if there is evidence of significant FI and prior conventional treatments have failed. Elevance Health captures 12% of the CM and 6% of the MAM. Notably, Elevance Health has no policy on CR after traumatic injury in 28 out of 50 states, while the remaining states also require documentation of significant FI. Centene Corp holds 14% of the Public Health Exchange market, primarily through Medicaid plans in all states. While Centene may cover reconstructive procedures for cosmetic deformities resulting from trauma, these policies are applied on a case-by-case basis in each state, resulting in a lack of standardization.

**Conclusions:**

Our analysis reveals a gap in insurance coverage for CR after burn injuries, with most providers excluding these treatments regardless of the trauma involved. The reliance on criteria linking coverage to FI creates barriers to essential treatments. Other conditions resulting in disfigurement, such as cancer, are required by law to provide coverage. Advocacy efforts and national policy reform are needed to ensure individuals affected by traumatic injuries can access necessary cosmetic interventions that improve their quality of life.

**Applicability of Research to Practice:**

The findings can guide advocacy for insurance policy reforms by highlighting the psychological and social impacts of burn scars, encouraging broader coverage beyond just functional impairment.

**Funding for the Study:**

N/A

